# Soret Effect and Chemical Process on MHD Oscillatory Flow in a Physiological Fluid

**DOI:** 10.1155/abb/8818822

**Published:** 2025-03-12

**Authors:** R. Kavitha, Nyagong Santino David Ladu, S. Ravi

**Affiliations:** ^1^Department of Mathematics and Statistics, Faculty of Science and Humanities, SRM Institute of Science and Technology, Kattankulathur Campus, Chengalpattu, India; ^2^Department of Mathematics and Physics, Rumbek University of Science and Technology, Rumbek, South Sudan; ^3^Department of Electronics and Communication Engineering, Saveetha School of Engineering, Saveetha Institute of Medical and Technical Sciences (SIMATS), Saveetha University, Chennai, Tamil Nadu, India

**Keywords:** chemical reaction, heat transfer, mass transfer, MHD, oscillatory flow, porous medium, Soret effect

## Abstract

This paper investigates the impact of chemical and Soret reactions on magnetohydrodynamic (MHD) oscillatory flow in a porous arteriole. Using appropriate mathematical techniques, a model of a mathematical equation is developed and solved. The flow governing equations are formulated based on certain assumptions. Exact solutions are attained for the profiles of velocity, temperature, and concentration. To highlight the key features, the numerical computations of the physical parameters, Grashof number, Reynolds number, Magnetic number, and Soret number were presented graphically. The present study reveals the viscoelasticity of blood significantly reduces flow velocity. And also illustrates blood flow (BF) in the artery is affected by the Lorentz force, which causes the velocity of the BF to increase as the magnetic field parameter values increase. The obtained outcome may be very useful in controlling BF during the surgical procedure.

## 1. Introduction

In the intricate landscape of fluid dynamics, the interaction between magnetic fields, chemical processes, and physiological fluids has emerged as a captivating area of exploration. This research endeavors to unravel the complexities inherent in the convergence of the Soret effect and chemical reactions within the framework of magnetohydrodynamic (MHD) oscillatory flow, particularly within physiological fluids. The significance of this study lies not only in its theoretical implications but also in its wide-ranging applications across several interdisciplinary domains. Physiological fluids, with their intricate composition and dynamic properties, present a fascinating milieu for scientific inquiry. Understanding the behavior of these fluids under the influence of external factors, such as magnetic fields and chemical reactions, is essential for advancing knowledge in fields ranging from biomedical engineering to environmental science. The Soret effect, characterized by thermal diffusion due to a gradient in temperature, introduces a unique dimension to fluid dynamics. When coupled with chemical processes and the application of magnetic fields, the applications and transformative insights.

In the realm of biomedical engineering and healthcare, this research holds promise for advancing our understanding of fluid dynamics within the human body. Insights gained may inform the design of medical devices and drug delivery systems, where precision and efficiency are paramount. The ability to control and predict oscillatory flow in physiological fluids can have direct implications for surgical procedures, offering new strategies for managing blood flow (BF) during interventions. Beyond healthcare, the study's findings may extend into environmental engineering, shedding light on fluid behavior in porous media affected by chemical reactions and magnetic fields. Moreover, applications in materials science and energy could benefit from a nuanced comprehension of how these factors influence fluid flow, with potential implications for optimizing materials and processes. As we embark on this exploration, the complex idea of the Soret effect, chemical processes, and MHD oscillatory flow promises not only to deepen our theoretical understanding but also to catalyze advancements with transformative applications. One intriguing aspect of this research lies in the exploration of MHD oscillatory flow phenomena, where the interaction between magnetic fields and fluid motion plays a pivotal role. MHD flows find applications in various fields, including medicine, where the dynamics of physiological fluids, such as blood and cerebrospinal fluid, are crucial for the proper functioning of the human body.

In recent years, researchers have delved into the complexities of MHD oscillatory flow within physiological fluids to unravel the underlying mechanisms and their potential implications for health and disease. Among the factors influencing such flows, the Soret effect and chemical processes emerge as key considerations that significantly impact the behavior of fluids under magnetic influence. The Soret effect, also known as thermophoresis, is a phenomenon wherein a temperature gradient induces mass transport in a fluid. Understanding the interplay between the Soret effect and MHD oscillatory flow becomes imperative for comprehending heat and mass transfer phenomena in physiological fluids. Additionally, chemical processes within the fluid add another layer of complexity as they can alter fluid properties and further influence the overall flow dynamics.

Blood is a complex fluid with non-Newtonian behavior. Unlike simple Newtonian fluids, blood exhibits shear-thinning behavior, meaning its viscosity decreases with increasing shear rate. This property is essential to capture accurately in modeling BF. BF in the human body encounters a wide range of shear rates in different vessels and under varying conditions. A non-Newtonian model better reflects the actual behavior of blood in these diverse physiological environments. Blood in arteries experiences oscillatory flow patterns due to the heartbeat.

This research aims to contribute to the growing body of knowledge in the field by investigating the combined effects of the Soret effect and chemical processes on MHD oscillatory flow within physiological fluids. The outcomes of this study have the potential to enhance our understanding of fluid dynamics in biological systems, with implications for medical diagnostics and treatment strategies. Furthermore, insights gained from this research may open avenues for optimizing the design of biomedical devices and technologies that interact with physiological fluids under the influence of magnetic fields.

The flow of blood through the arterial system of a human being is treated as the application of a fluid dynamics problem. Researchers will gain a better insight into the human body's physiology by stimulating the BF rate in each segment of the arterial network system. As a result of the development of arterial stenosis—hemodynamics plays a vital role, which leads to cardiovascular system dysfunction. The majority of deaths in developed countries are caused by cardiovascular problems. Earlier, elderly people were the most affected by cardiovascular disease, but this is no longer the case. Age, gender, tobacco use, high blood pressure, and cholesterol, among other factors, all contribute to the development of stenosis. Stenosis is a type of blood vessel occlusion caused by the buildup of cholesterol, fat, and abnormal tissue growth at the arterial wall. Enlarging the stenosis, leading to an increase in cardiovascular diseases such as heart attack, strokes, etc. In some circumstances, the influence of density variations of the blood with concentration and temperature can be coupled. When the Soret and Dufour effects are significant, direct coupling of concentration and temperature is possible in some cases. From temperature, mass flux is calculated because of the Soret effect, and from the concentration, energy flux is predicted because of the Dufour effect.

Sinha and Misra [1] investigated the flow of blood through a porous blood vessel component in the existence of a magnetic field. He explored Variable hematocrit, viscosity variation, and velocity slip at the arterial wall. To carry out research, a model based on MHD principles was developed. The authors created analytical expressions for the velocity of blood, wall shear stress, and pressure gradient. Tzirtzilakis [2] investigated biomagnetic fluid dynamics BFD problems. He described blood as the Newtonian fluid. He followed the principles of Ferro-hydrodynamics and MHDs, and it accounts for blood magnetization as well as electrical conductivity. The numerical study of Newtonian biomagnetic fluid blood in the impact of a magnetic field was described by the author. Sharma, Jain, and Kumar [3] investigated two types of blood rheological models: the power law and generalized Maxwell models. They investigated the impact of a magnetic field in this model. Sharma's study described these theological properties that influence BF and hemodynamics. This could be extremely helpful for doctors in predicting diseases based on the flow pattern for an elastic artery insight of a magnetic field. Thomas and Sumam [4] investigated the overview of the structure and functions of arteries and veins. BF models and the structure of fluid interaction in the artery were also investigated.

Abubakar and Adeoye [5] explored the influence of a magnetic field and radiation in a stenosed artery. They also considered blood as the Newtonian fluid. The authors used the differential transform method (DTM) under mild stenosis. Prakash et al. [6] studied MHD BF along bifurcated arteries in the existence of a heat source. Here authors have taken blood through arteries is unsteady Newtonian fluid flow. Kumar et al. [7] investigated MHD BF in permeable arteries in the existence of heat and chemical reactions. The conclusion taken from this study is used in carotid body cancer treatment that occurs in bifurcated arteries. Mekheimer, Haroun, and Elkot [8] depicted the flow of blood along an artery, which is elastic with multistenosis. To simulate the arterial segment, an anisotropically cylindrical tube, which is elastic and pervaded with a viscous fluid representing blood, was used. Nadeem et al. [9] investigate the biomagnetic fluid flow along with stenosis tapered porous artery. Here, blood is treated as a Newtonian biomagnetic fluid. Kumar [10] studied that there exists a lubricating layer between red blood cells (RBCs) and the tube wall. In his study, he has added the effect of a porous medium. Tripathi and Sharma [11] focused on an inclined porous stenosed artery, introducing complexity to the model as it reflects the realistic geometrical features of stenosed arteries. El-Khatib et al. [12] presented models depending on the level of cholesterol (LDL). As the disease progresses, atherosclerotic plaque forms, vessels remodel, and the plaque may fracture due to its interplay with BF. They discussed plaque–flow interaction models and reduced BF models in atherosclerotic vasculature (0D and 1D).

Kumar [13] investigated the development of a method for modeling through an atherosclerosis-affected region on pulsating BF in the artery by finite difference method, as well as the statistical model. Priyadharshini and Poalagusamy [14] investigated the presence of periodic acceleration of the human body in the presence of MHD flow parameters of the blood. Blood is assumed to behave like a Casson fluid. Authors have used finite difference schemes to find solutions for the mathematical model. Misra and Adhikary [15] investigated oscillatory MHD BF in a single framework effect of the chemical reaction. They treated blood as a second-grade fluid.

Sasikumar and Govindarajan [16] discussed the Soret effect on MHD oscillatory flow with a heat source through a porous medium in an asymmetric wavy channel. They suggested an exploration of the influence of chemical reactions and thermal radiation on fluid flow in complex geometries. Noor et al. [17] investigated the combined effects of Soret and Dufour effects on the MHD squeezing flow of Jeffrey fluid in a horizontal channel with thermal radiation. This paper indicates a study on how various physical parameters affect the behavior of non-Newtonian fluids under different flow conditions. Hayat et al. [18] explored the effects of convective conditions and chemical reactions on the peristaltic flow of Eyring–Powell fluid. Peristaltic flow models are often used to study the motion of biological fluids through tubular structures, and this paper likely delves into how these processes are influenced by chemical reactions. Vijayakumar et al. [19] investigate the role of Soret and Dufour effects on unsteady MHD oscillatory Casson fluid flow on an inclined vertical porous plate in the presence of a chemical reaction.

Abonyo and Awuor [20] examine the effects of thermal radiation and chemical reactions on hydromagnetic fluid flow in a cylindrical collapsible tube with an obstacle. Kala and Rawat [21] studied the effect of chemical reactions and oscillatory suction on MHD flow through porous media in the presence of pressure. This likely explores how chemical reactions and external forces influence the flow of conducting fluids through porous materials. Mehrabi and Satayeshi [22] focused on pulsatile BF behavior in modeled stenosed vessels with different severities. This paper contributes to the understanding of BF patterns and behaviors in narrowed blood vessels, which is crucial for understanding cardiovascular diseases. Kamada et al. [23] discussed BF analysis using 4D flow MRI for vascular diseases. This paper likely explores the integration of advanced imaging techniques and computational modeling to better understand and diagnose vascular pathologies. Sarwar et al. [24] investigated the thermal enhancement and numerical solution of blood nanofluid flow through a stenotic artery. The authors used nanofluids and heat transfer enhancement techniques to improve BF in diseased arteries. Shah and Kumar [25] explored the mathematical modeling of BF with the suspension of nanoparticles through a tapered artery with a blood clot. This paper likely explored the use of nanoparticles for targeted drug delivery and therapeutic interventions in cardiovascular diseases.

In the field of fluid dynamics, the study of oscillatory flow in physiological fluids is of great importance due to its relevance in various biological and medical applications, such as BF in the circulatory system and airflow in the respiratory system. The use of a magnetic field in this context, known as MHDs, adds a new dimension to the study by considering the interaction between the fluid flow and magnetic field. Previous research has focused on the application of magnetorheological fluids in systems such as building structures and automobiles to improve performance and reduce vibration. However, the application of magnetorheological fluids in physiological fluids, specifically in oscillatory flow, is relatively novel and presents new opportunities for research and innovation. In this paper, we aim to investigate the Soret effect and chemical processes in MHD oscillatory flow in a physiological fluid. The focus will be on understanding how the presence of a magnetic field affects the transport of species and the occurrence of chemical reactions in oscillatory flow. Understanding these phenomena is crucial for the development of biomedical devices and therapies that involve oscillatory flow, as the interaction between the magnetic field and physiological fluids can significantly impact the effectiveness and safety of these systems.

In response to the preceding analysis, this paper examines MHD BF along a stenosed inclined porous artery in the influence of the Soret effect and heat source. Under well-defined boundary conditions, the governing equations of BF are solved. Using the closed-form perturbation method, the model of the mathematical problem is discovered and solved. Closed-form solutions provide explicit mathematical expressions that allow for a clear and direct understanding of the relationships between variables. This transparency facilitates a straightforward interpretation of the effects of various parameters on the system, enhancing the clarity of the analysis. Graphs depict the impacts of various physical parameters on the velocity and temperature profile of BF, such as artery inclination angle, porosity, magnetic field, and heat source parameters.

## 2. Mathematical Analysis

**Figure 1:**
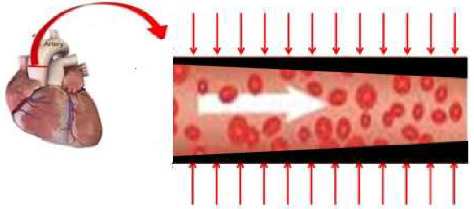
Blood flow inside the artery when constant heat flux is applied.

Blood exhibits non-Newtonian behavior, meaning its viscosity is not constant but varies with the shear rate. At low shear rates, such as those experienced in small vessels and capillaries, blood behaves more like Newtonian fluid. However, at higher shear rates, as seen in larger vessels, the viscosity of blood decreases, and it demonstrates shear-thinning behavior. The non-Newtonian nature of blood is mainly attributed to the presence of RBCs (erythrocytes) and their interactions with plasma. The deformability of RBCs and their tendency to aggregate in certain conditions contribute to the non-Newtonian rheological properties of blood.  Figure 1 shows the BF inside the artery, the time when constant heat flux is applied.

This model is for a pathological condition of an arterial in which the lumen has become porous due to the deposition of various materials such as cholesterol, lipids, and fatty substances. The term “blood” refers to a plasma-based suspension of erythrocytes and other microelements. The assumption is that blood is uniformly dense in the segment under consideration. In the *y*-direction, a magnetic field with constant intensity is assumed to be applied. The primary causes of convection that are being studied are temperature and concentration variations. The rheology of blood flowing through a permeable bifurcated artery influence of chemical and heat sources is mathematically modeled.

Momentum Equation

(1) $$\begin{array}{c} \frac{\partial u}{\partial t}=-\;\frac{1}{\rho }\frac{\partial P}{\partial x}+g\beta^{*}\left(C-C_{0}\right)+\gamma \frac{\partial^{2}u}{\partial y^{2}}-\frac{\sigma {B_{0}}^{2}}{\rho }u-\frac{\gamma }{k}u+g\beta \left(T-T_{0}\right)+\beta \left(\frac{\partial^{3}u}{\partial y^{2}\partial t}\right). \end{array}$$

Energy Equation

(2) $$\begin{array}{c} \frac{\partial T}{\partial t}=\frac{\kappa }{\rho C_{p}}\frac{\partial^{2}T}{\partial y^{2}}+\frac{1}{\rho C_{p}}\;\frac{\partial q_{r}}{\partial y}+\frac{Q}{\rho C_{p}}. \end{array}$$

Concentration Equation

(3) $$\begin{array}{c} \frac{\partial C}{\partial t}=D_{M}\frac{\partial^{2}C}{\partial y^{2}}+\frac{Dk_{T}}{T_{m}}\frac{\partial^{2}T}{\partial y^{2}}-K_{c}^{\prime }\left(C-C_{0}\right), \end{array}$$

with the boundary conditions,

(4) $$\begin{array}{c} u=\lambda \frac{\partial u}{\partial y},\;T=\;T_{0}+\left(T_{w}-T_{0}\right)e^{i\omega t},\;C=C_{0}+\left(C_{w}-C_{0}\right)e^{i\omega t}\text{ at }y=h, \end{array}$$

(5) $$\begin{array}{c} u=\lambda \frac{\partial u}{\partial y},\;T=\;T_{0},\;C=C_{0}\text{ at }y=0. \end{array}$$

The radiative transfer term is given using the Rosseland approximation,

(6) $$\begin{array}{c} q_{r}=-\frac{4\sigma }{3\propto }\frac{\partial T^{4}}{\partial y}, \end{array}$$

where

(7) $$\begin{array}{c} T^{4}=4T_{0}^{3}T-3T_{0}^{4}. \end{array}$$

Then heat transfer equation becomes,

(8) $$\begin{array}{c} \frac{\partial T}{\partial t}=\frac{\kappa }{\rho C_{p}}\frac{\partial^{2}T}{\partial y^{2}}-\frac{16\sigma T_{0}^{3}}{3\alpha \rho C_{p}}\;\frac{\partial^{2}T}{\partial y^{2}}+\frac{Q}{\rho C_{p}}. \end{array}$$

Dimensionless variables are,

(9) x→= xh; y→= yh; u→= uU; Re=Uhγ; T=T−T0Tw−T0; C=C−C0Cw−C0; M2=σB02h2ργ; t→= tUa; P→= PhργUDa= kh2; Da= kh2;s2=1Da; Sc=DMUh; Sr= DkTTmUhTw−T0Cw−C0; Gr= gβTw−T0γUh2; Gc=gβ∗Cw−C0γU h2Pr= ρCpk; N2=4α2a2k; Kc=DKc⁣′Cw−C0; α= Qh2KTw−T0; ω=ωhU.

### 2.1. Solution of the Problem

Obtain closed-form solutions in the study of the Soret effect and chemical processes on MHD oscillatory flow in a physiological fluid, certain assumptions are likely made to simplify the mathematical model. While the specific assumptions can vary based on the details of the study, here are some common assumptions that are often made in such analysis:

Assuming steady-state conditions or simplifying the transient terms in the governing equations may be done to simplify the mathematical analysis. This is a common approach to obtaining closed-form solutions, but it may limit the model's representation of dynamic or time-dependent behaviors. Linearizing certain terms in the equations simplifies the mathematical model, making it amenable to closed-form solutions. This is often used to handle nonlinearities and may be suitable within a limited range of conditions. Omitting external forces such as gravity or other body forces can simplify the equations. This might be done to focus on the core dynamics of the system and obtain a closed-form solution without the added complexity of external influences.

After removing the bars, the solution to the problem be,

(10) $$\begin{array}{c} Re\frac{\partial u}{\partial t}=\;-\frac{\partial P}{\partial x}+\frac{\partial^{2}u}{\partial y^{2}}-\left(S^{2}+M^{2}\right)u+Gr\theta +GcC+\beta \left(\frac{\partial^{3}u}{\partial y^{2}\partial t}\right), \end{array}$$

(11) $$\begin{array}{c} Pr\frac{\partial \theta }{\partial t}=\;\frac{\partial^{2}\theta }{\partial y^{2}}+\left(N+\alpha \right)\theta , \end{array}$$

(12) $$\begin{array}{c} \frac{\partial C}{\partial t}=Sc\;\frac{\partial^{2}C}{\partial y^{2}}+Sr\;\frac{\partial^{2}\theta }{\partial y^{2}}-K_{c}C, \end{array}$$

with the boundary conditions,

(13) $$\begin{array}{c} u=\lambda \frac{\partial u}{\partial y},\;\theta =\;e^{i\omega t},\;C=e^{i\omega t}\text{ at }y=1, \end{array}$$

(14) $$\begin{array}{c} u=\lambda \frac{\partial u}{\partial y},\;\theta =\;0,\;C=0\text{ at }y=0. \end{array}$$

### 2.2. Method of Solution

Assuming a pressure gradient for purely oscillatory flow as follows:

(15) $$\begin{array}{c} -\frac{\partial P}{\partial x}=\lambda e^{i\omega t}, \end{array}$$

where *λ* is constant and *ω* is the frequency of oscillations. Assume that the solutions for *u*(*y*, *t*),  *θ*(*y*, *t*),  and *C*(*y*, *t*) are in the form,

(16) $$\begin{array}{c} \left.\begin{array}{c} u\left(y,t\right)=\;u_{0}\left(y\right)e^{i\omega t}\\ \theta \left(y,t\right)=\;\theta_{0}\left(y\right)e^{i\omega t}\\ C\left(y,t\right)=\;C_{0}\left(y\right)e^{i\omega t} \end{array}\right\}. \end{array}$$

Substituting Equations (15) and (16) in Equations (10)–(12), we get,

(17) $$\begin{array}{c} \frac{d^{2}\theta_{0}}{dy^{2}}+m_{1}\theta_{0}=0, \end{array}$$

(18) $$\begin{array}{c} \frac{d^{2}C_{0}}{dy^{2}}-{m_{2}}^{2}C_{0}=\;\frac{Sr}{Sc}\;{m_{1}}^{2}\theta_{0}, \end{array}$$

(19) $$\begin{array}{c} \left(1+\beta i\omega \right)\frac{d^{2}u_{0}}{dy^{2}}-{m_{3}}^{2}u_{0}=-\lambda -Gr\theta_{0}-GcC_{0}, \end{array}$$

with the boundary conditions,

(20) $$\begin{array}{c} u_{0}=\lambda \frac{\partial u_{0}}{\partial y},\;\theta_{0}=\;1,\;C_{0}=1\text{ on }y=1, \end{array}$$

(21) $$\begin{array}{c} u_{0}=\lambda \frac{\partial u_{0}}{\partial y},\;\theta_{0}=\;0,\;C_{0}=0\text{ on }y=0, \end{array}$$

where $m_{1}=\;\sqrt{N+\alpha -Pri\omega },m_{2}=\;\sqrt{\frac{K_{c}+i\omega }{Sc}}$ and $m_{3}=\;\sqrt{S^{2}+M^{2}+i\omega Re}$.

Equations (17)–(19) are solved using Equations (20) and (21), we obtain,

(22) $$\begin{array}{c} \theta \left(y,t\right)=\;\frac{\sin m_{1}y}{\sin m_{1}}\;e^{i\omega t}\;, \end{array}$$

(23) $$\begin{array}{c} \phi \left(y,t\right)=\;\left[\frac{\sin hm_{2}y}{\sin hm_{2}}+\frac{Sr}{Sc}\;\frac{m_{1}}{\left({m_{1}}^{2}+{m_{2}}^{2}\right)}\;\frac{\sin hm_{2}y}{\sin hm_{2}}-\;\frac{Sr}{Sc}\;\frac{m_{1}}{\left({m_{1}}^{2}+{m_{2}}^{2}\right)}\frac{\sin hm_{1}y}{\sin hm_{1}}\right]e^{i\omega t}\;, \end{array}$$

(24) $$\begin{array}{c} u\left(y,t\right)=\left[Ee^{m_{4}y}+Fe^{-m_{4}y}+\frac{\lambda }{Rei\omega +S^{2}+M^{2}}+\frac{Gr}{e^{m_{1}}-e^{-m_{1}}}\left[\frac{e^{m_{1}y}}{X}-\frac{e^{-m_{1}y}}{X}\right]+\frac{Gc}{e^{m_{2}}-e^{-m_{2}}}\left[\frac{e^{m_{2}y}}{Y}-\frac{e^{-m_{2y}}}{Y}\right]\right]e^{i\omega t}\;. \end{array}$$

(All parameter expansions are provided in the Appendix).

## 3. Results and Discussions

**Figure 2:**
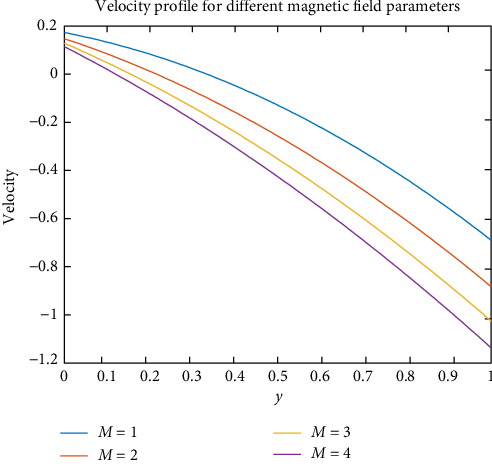
Effect of magnetic field on velocity.

For various parameters, the distribution of different profiles has been studied.  Figure 2 depicts the effect of magnetic field parameters on BF velocity profiles. As the magnetic field increases, the velocity of BF decreases. It occurs when the flow of blood is affected by a magnetic field. Magnetization effort causes the charged particles in the blood to rotate. RBCs become more draped in blood plasma due to the continuous motion of charged particles, and the blood's internal viscosity increases. Increased viscosity reduces BF by increasing the value of resistance flow, resulting in blood velocity decreases. Lorentz force opposes blood particle motion, lowering BF velocity.

**Figure 3:**
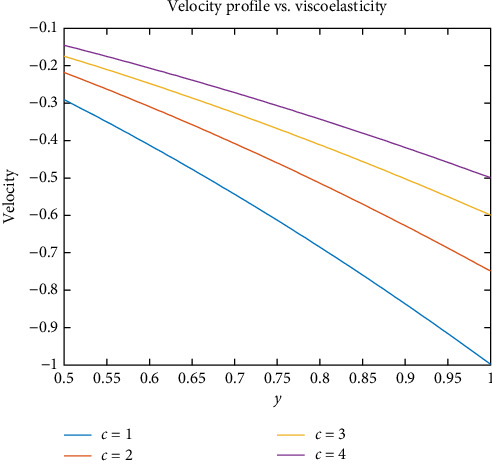
Effect of viscoelasticity on velocity.

Figure 3 indicates velocity of blood decreases as the viscoelasticity of blood increases. It shows that viscoelasticity increases and velocity decreases. Because blood's viscosity increases at low flow rates, in this state, between red cells and plasma proteins, more molecular interactions will occur. This will increase the viscosity of blood because of the more molecular interactions between red cells and plasma. This will give rise to these red cells and allow them to stick together and create chains of multiple cells within the microcirculation. Due to this high-level element's interaction, the blood becomes stationary—not flowing. This type of blood requires pressure to make the BF.

**Figure 4:**
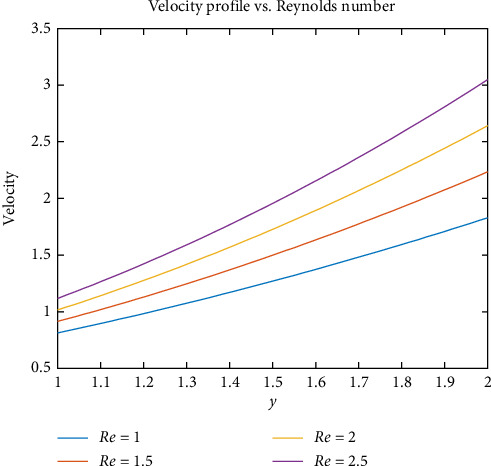
Effect of Reynolds number in velocity.

**Figure 5:**
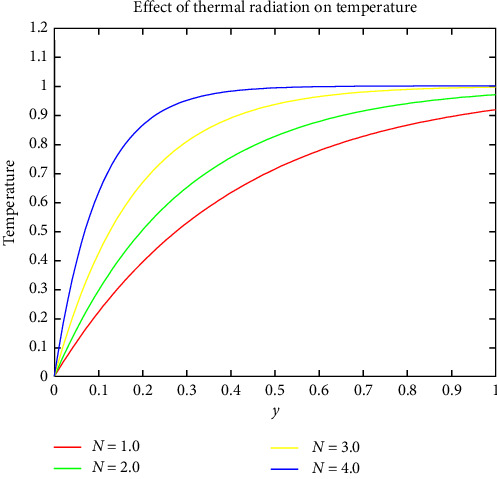
Effect of thermal radiation on temperature.

Figure 4 shows the fluctuation of the BF velocity profile for various Reynolds numbers. The Reynolds number is proportional to the density of the blood, the velocity of blood, and the diameter of the blood vessel. Increasing the magnitude of any of these parameters raises the Reynolds number.  Figure 5 depicts the variation of the BF temperature profile for different values of thermal radiation. Radiation acts as a source of heat within the blood. As a result, increasing the radiation dosage increases the BF temperature profile directly. This type of thermal therapy is commonly used in the pathological state to subject body tissues and cancerous tumors to high temperatures. As a result, cancer cells associated with tumors are killed, while normal tissues suffer minimal damage.

**Figure 6:**
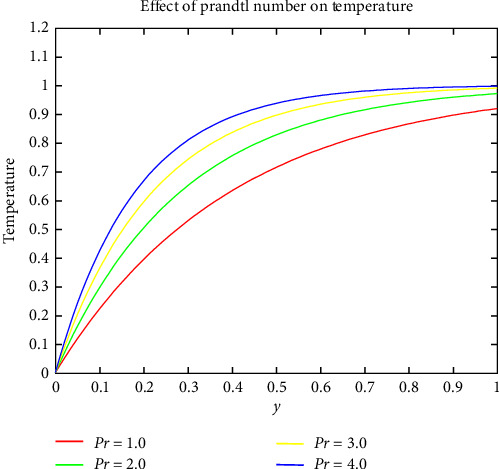
Effect of Prandtl number on temperature.

**Figure 7:**
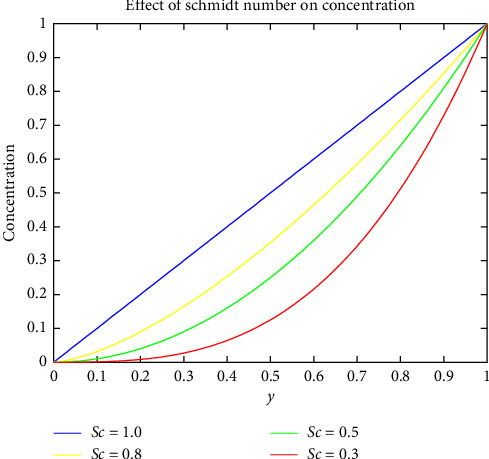
Effect of Schmidt number on concentration.

**Figure 8:**
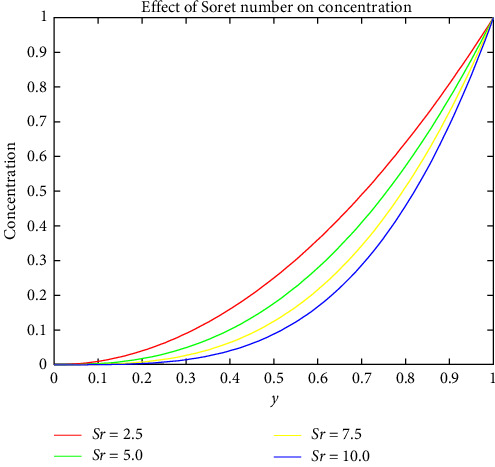
Effect of Soret number on concentration.

Figure 6 shows how the Prandtl number varies with temperature. The Prandtl number's value is proportional to the thickness of the blood's boundary layer. Increasing the magnitude of the Prandtl parameter raises the temperature profile. When the Prandtl number is increased, it implies that thermal diffusivity becomes more dominant compared to kinematic viscosity. This indicates that the fluid is more effective at conducting heat. Higher Prandtl numbers are associated with thinner thermal boundary layers, facilitating more efficient heat conduction across the fluid and contributing to an elevated temperature profile. Figures 7 and 8 indicate the effect of the Schmidt and Soret numbers on the field of concentration. It is noticed that the increase in Schmidt number drastically increases the concentration due to the porosity within the hydrodynamic and boundary layer. An increase in Schmidt number implies that momentum diffusivity is relatively higher compared to mass diffusivity. In the context of mass transfer, a higher Schmidt number indicates that the fluid has greater resistance to the diffusion of mass. And she also observed that the Soret number and concentration are directly propositional to one another. As the Soret number increases, it indicates that thermal diffusion becomes more dominant. The direct proportionality between the Soret number and concentration could be attributed to the influence of temperature gradients on mass diffusion. When the Soret number is higher, the impact of thermal gradients becomes more pronounced, affecting the distribution and transport of mass within the fluid.

**Figure 9:**
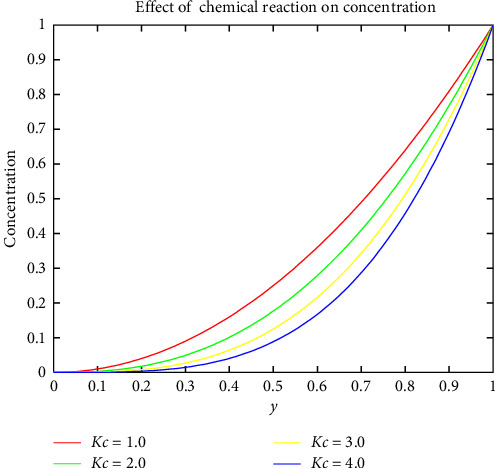
Effect of chemical reaction on concentration.

**Figure 10:**
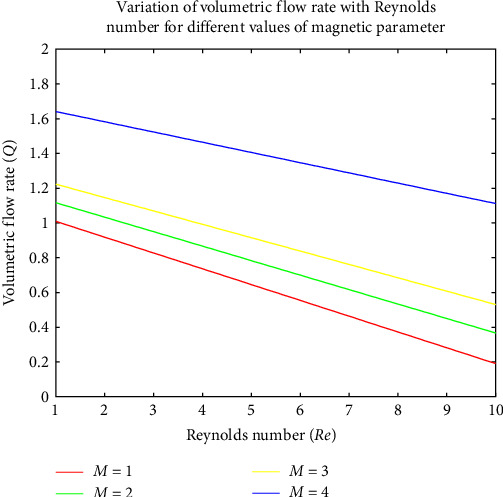
Variation of volumetric flow rate with Reynolds number for different values of magnetic parameter.

Figure 9 indicates the impact of the chemical process on the concentration. It is observed that the chemical process diminishes the concentration, resulting in a thinner boundary layer. If the chemical process decreases the dynamic viscosity of the fluid, it can lead to a thinner boundary layer. This is because lower-viscosity fluids tend to exhibit reduced resistance to shear, resulting in thinner boundary layers.  Figure 10 shows that the Reynolds number increases, the value of the MHD intensity and the volumetric flow rate decrease, and the variation occurs almost rectilinearly for all magnetic parameters. It is also discovered that increasing the magnetic factor *M* from 1 to 4 significantly reduces the volumetric flow rate.

## 4. Conclusion

This study presents a comprehensive investigation of the effects of chemical reactions, particularly the Soret effect, on MHD oscillatory BF in a porous arterial segment caused by fatty substance deposition. The flow dynamics are thoroughly analyzed, leading to the following key conclusions:

• The viscoelastic properties of blood significantly reduce flow velocity.
• The Lorentz force influences BF within the artery, reducing velocity as the magnetic field strength increases. These findings may have critical applications in regulating BF during surgical procedures.
• BF velocity decreases as viscoelasticity increases due to the higher density of RBCs in the artery, which impedes the flow.
• Radiation acts as a heat source in blood, directly increasing the BF temperature profile with higher radiation doses.
• The Schmidt and Soret numbers are found to have a direct proportional relationship with the concentration field.
• As the Reynolds number increases, the intensity of MHD effects and the volumetric flow rate decrease. Fluctuations in these parameters exhibit nearly rectilinear behavior across all magnetic parameter values considered in the study.

## Data Availability

The data sets used and analyzed during the current study are available upon reasonable request from the corresponding author.

## Appendix

• ***X* = (1 + *βiω*)*m*_1_^2^ − (*Reiω* + *S*^2^ + *M*^2^).**
• ***Y* = (1 + *βiω*)*m*_2⁣_^2^ − (*Reiω* + *S*^2^ + *M*^2^).**
• $m_{1}=\;\sqrt{N+\alpha -Pri\omega }$.
• $m_{2}=\;\sqrt{\frac{K_{c}+i\omega }{Sc}}$.
• $m_{3}=\;\sqrt{S^{2}+M^{2}+i\omega Re}$.
• $m_{4}=\sqrt{\frac{i\omega Re+S^{2}+M^{2}}{1+\beta i\omega }}$.
• $Da=\;\frac{k}{h^{2}}$.
• $s^{2}=\frac{1}{D_{a}}$.
• $Sc=\frac{D_{M}}{Uh}$.
• $Sr=\;\frac{Dk_{T}}{T_{m}Uh}\left(\frac{T_{w}-T_{0}}{C_{w}-C_{0}}\right)$.
• $Gr=\;\frac{g\beta \left(T_{w}-T_{0}\right)}{\gamma U}h^{2}$.
• $Gc=\frac{g\beta^{*}\left(C_{w}-C_{0}\right)}{\gamma U}\;h^{2}$.
• $Pr=\;\frac{\rho C_{p}}{k}$.
• $N^{2}=\frac{4\alpha^{2}a^{2}}{k}$.
• *K*_*c*_ = *DK*_*c*_′(*C*_*w*_ − *C*_0_).
• $\alpha =\;\frac{Qh^{2}}{K\left(T_{w}-T_{0}\right)}$.
• $\omega =\frac{\omega h}{U}$.
• $A=\frac{\lambda }{Rei\omega +S^{2}+M^{2}}+\frac{Gr}{e^{m_{1}}-e^{-m_{1}}}\left[\frac{\left(1-\lambda m_{1}\right)e^{m_{1}}}{X}-\frac{\left(1+\lambda m_{1}\right)e^{-m_{1}}}{X}\right]+\frac{Gc}{e^{m_{2}}-e^{-m_{2}}}\left[\frac{\left(1-\lambda m_{2}\right)e^{m_{2}}}{Y}-\frac{\left(1+\lambda m_{2}\right)e^{-m_{2}}}{Y}\right].$
• $B=\frac{\lambda }{Rei\omega +S^{2}+M^{2}}+\frac{Gr}{e^{m_{1}}-e^{-m_{1}}}\left[\frac{\left(1-\lambda m_{1}\right)}{X}-\frac{\left(1+\lambda m_{1}\right)}{X}\right]+\frac{Gc}{e^{m_{2}}-e^{-m_{2}}}\left[\frac{\left(1-\lambda m_{2}\right)}{Y}-\frac{\left(1+\lambda m_{2}\right)}{Y}\right]$.
• $E=\frac{A-Be^{-m_{4}}}{\left(e^{m_{4}}-e^{-m_{4}}\right)\left(1-\lambda m_{4}\right)}$.
